# The impact of multiple discrimination on labor misallocation of China: Based on fsQCA method

**DOI:** 10.1371/journal.pone.0308442

**Published:** 2024-08-06

**Authors:** Rongwang Guo, Jianxiu Wang, Yuntian You

**Affiliations:** School of Management Science and Engineering, Shanxi University of Finance and Economics, Taiyuan, China; Beijing University of Technology, CHINA

## Abstract

Discrimination in the labor market hinders efficient labor allocation, impeding socio-economic health. With the rapid population aging in China, addressing multifaceted discrimination to enhance labor allocation efficiency emerges as a crucial area of research. To explore the relationship between five types of discrimination (age, gender, hukou, educational background, and occupation) and labor misallocation, this paper based on intersectionality theory, employs the fuzzy-set Qualitative Comparative Analysis (fsQCA) method to conduct a configurational analysis of data from China. The research findings indicate that none of the five forms of discrimination can be deemed a necessary condition for achieving high-level labor misallocation. The study identifies five distinct pathways of multiple discrimination to form high-level labor misallocation, which can be classified into four interaction modes: age-hukou, gender-hukou, gender-occupation, and age-gender-educational background. Meanwhile, there are four configuration paths for the absence of labor misallocation. This study reveals the intricate mechanisms by which multiple forms of discrimination contribute to labor misallocation in China’s labor market, and provides valuable insights for addressing employment discrimination and improving the efficiency of labor allocation.

## 1. Introduction

Under the global trend of an aging population, the effective allocation of labor factors is pivotal for economic growth across the globe. China, especially as a major world population country, has the highest rate of population aging in the world, leading to rapid transformative change in its labor market structure. This could result in labor shortages and slowed economic growth. Therefore, enhancing the allocation efficiency of labor factors is crucial for the high-quality development of China’s economy.

In previous studies, the determinants of labor allocation efficiency are broadly categorized into three main dimensions: institutional factors, market mechanisms, and technological advancements. The institutional factors include industrial policies [1], market segmentation [2], government audit [3], and hukou reform [4]. On the market side, financial frictions [5], labor market rigidities [6], and land transfer mechanisms [7] are key elements that can impede or facilitate efficient labor allocation. The technological advancements include the internet [8], digital technology [9], and artificial intelligence [10]. Although employment discrimination is believed as a barrier to the development of the labor market, few scholars have directly investigated its impact on the efficiency of labor allocation. Thus, how to further eliminate employment discrimination to improve the efficient allocation of labor factors is still an important question underexplored.

Discrimination matters in the labor market, which undermines the legitimate rights and interests of workers and impedes the efficient allocation of labor factors [11,12]. Actually, employment discrimination is a complex issue due to its entanglement with the intersecting identities of individuals, such as race, gender, age, and educational background. These intersecting factors often subject workers to the cumulative impact of multiple forms of discrimination [13,14]. Especially over the past decades, as the intersectionality theory has developed, the ever-increasing scholars appeal to examine interactions between dimensions of identity beyond just race and gender [13,15]. The concept of intersectionality refers to the understanding that an individual’s race, gender, age, educational background, and other personal characteristics are not single or mutually exclusive, but rather interdependent phenomena that contribute to the formation of complex social inequalities [14–16]. In recent years, intersectionality has become the most widely used framework in feminist theory research. Scholars within traditional social science disciplines, as well as those working within more applied fields such as public policy, psychology, epidemiology, education, public policy, and criminology, have found intersectionality to be of value [13,15].

However, existing literature on the negative impact of discrimination mainly focused on the workers’ job opportunities [17] and wages [18]. Only several scholars have conducted macro-level studies on the effects of discrimination. Biddle and Hamermesh (2013) tested the relationship between discrimination and economic cycles empirically, and found that the gender wage gap is counter-cyclical, with women experiencing a relative wage disadvantage during economic downturns (high unemployment rates). Conversely, the discriminatory gap among African-Americans is pro-cyclical [19]. Li (2019) further verified that gender discrimination has a negative effect on firms’ total factor productivity [20]. Li’s research showed that firms with a higher number of female employees tend to have greater total factor productivity. Clearly, these studies primarily focus on the impact of single types of discrimination on individual workers, with no research undertaken on the relationship between discrimination and labor allocation efficiency. Moreover, research on factors influencing labor distribution efficiency has typically focused on the net effects of relationship. Existing research has not captured the complexity of multiple discriminations in the labor market, as labor misallocation may be subject to a cumulative effect of discrimination such as age, gender, and household registration. Thus, there is a research gap in revealing the complex mechanisms by which multiple discriminations affect labor misallocation.

In order to thoroughly explore the relationship between multiple discrimination and labor misallocation, this study establishes a comprehensive analytical framework based on the intersectionality theory, encompassing five types of discrimination: age, gender, hukou, educational background, and occupation. Considering the limitations of traditional linear analysis methods in exploring the complex causal relationships between variables [13,21], this study employs fuzzy-set Qualitative Comparative Analysis (fsQCA) to reveal the underlying complex mechanisms connecting discrimination and labor misallocation. And the data from the Chinese Social Survey 2021 and the Statistical Yearbook of China. This paper addresses the research gap in the study of labor allocation efficiency from a complexity perspective, providing a solid theoretical foundation for the government to formulate relevant policies. This study primarily aims to provide guidance for the elimination of various forms of discrimination within the Chinese labor market and to enhance the efficiency of labor allocation. By integrating intersectionality theory with fsQCA, this study has provided a detailed exposition of the mechanisms by which multiple discriminations affect labor market misallocation, offering a novel approach for similar research endeavors.

## 2. Theoretical framework

### 2.1. Intersectionality

During the 1960-1970s, the waves of Black feminist rights movements continued to surge in the U.S. Black women faced extreme oppression because they were both black and female, which promoted the reform and development of feminist theory. In 1989, legal scholar Crenshaw introduced the term "intersectionality" to better explain the interaction between various forms of oppression faced by African-American women [22]. She argued that discrimination is a complex phenomenon resulting from the intersection of their various identities, rather than being attributed to any single form of discrimination or a simple combination of various forms of discrimination. For instance, the experiences of Black women could not be accurately described through the perspectives of either white women or black men. After that, Collins (2000) further expanded on the concept by capturing the manifestations and interactions of multiple forms of discrimination within cultural and social systems, which laid the groundwork for the early understanding of intersectionality in politics and society [23].

The key features of intersectionality are simultaneous existence, multidimensionality, and cumulative effect [13,15,23]. This perspective opposes the simplistic aggregation of different forms of identity-based discrimination or disadvantages. Accordingly, some scholars have attempted to examine inequalities within intersectionality by integrating complexity theory with the concept of intersectionality [24,25]. Intersectionality can be explored as a research subject to investigate the social status and rights inequalities resulting from the intersection of different identities. It can also serve as a new theoretical framework for analyzing complex social phenomena [15,16]. When utilized as a theoretical framework, intersectionality requires quantitative researchers to avoid adopting a linear and singular perspective in both results and processes. Instead, they should construct theoretical explanations that align with the intricate realities of societal situations [13,16].

Additionally, in international recommendations for eliminating various forms of discrimination, the concept of intersectionality is also being introduced [26]. From a legal perspective, the purpose of intersectionality is to protect complex situations that cannot receive adequate justice when each form of discrimination is addressed separately by legal norms [27]. Consequently, the application of intersectionality theory can assist policymakers in better promoting equity-based improvements and social justice within a society that is becoming increasingly complex and diverse [28]. However, this theory has not been widely applied in the study of employment discrimination in China’s labor market.

### 2.2. Discrimination

The International Labor Organization (ILO) regards discrimination as any distinction, exclusion, or preference based on race, color, sex, religion, political opinion, national extraction, or social origin, which has the effect of denying or impairing equality of opportunity or treatment in employment or occupation [29]. The earliest research on discrimination in the labor market can be traced back to Becker’s (1971) economic model of discrimination, which investigated employer discrimination, employee discrimination, and customer discrimination [30]. Following Becker, numerous studies have explored various types of discrimination in the labor market. In the labor market of China, the primary forms of discrimination include age, gender, hukou, educational background, and occupation [31–33]. Among them, hukou discrimination is a unique form under China’s hukou system, which plays a significant role in determining an individual’s access to social services, benefits, and employment opportunities.

Discrimination has profound and varied consequences within the labor market, creating unequal employment opportunities that prevent workers from securing suitable positions and increasing job transition and search costs [17]. It also disrupts wage levels, upsetting the labor market’s supply-demand equilibrium, and generates wage disparities [18]. Furthermore, workers may also face issues such as wage arrears, unpaid overtime, employer abuse, and unhealthy working environments [34]. When workers perceive unfair treatment, they may find their fundamental needs unmet, potentially leading to adverse effects on their physical and mental health [35]. This perceived discrimination not only diminishes job satisfaction and security but also raises the risk of anxiety and depression [36]. Thus, employee motivation may wane, leading to counterproductive behaviors like tardiness, early departure, negligence, and absenteeism that diminish productivity. More severely, discrimination can lead to social exclusion and anti-discrimination actions among workers [37]. As a consequence, discrimination leads to the waste and loss of labor resources, thereby reducing labor output [38] and resulting in insufficient efficiency in labor allocation.

### 2.3. Analytical framework

The analysis presented indicates that discrimination leads to labor misallocation. While a single form of discrimination can disrupt the efficient allocation of labor resources, the compound effects of multiple discriminations could potentially have a more complex impact on the labor market dynamics. Drawing on intersectionality theory, multiple forms of discrimination in the labor market are simultaneous and exhibit cumulative effects [14,15]. In other words, multiple discrimination has a more severe impact on labor allocation efficiency compared to single discrimination. In view of this, it is essential for research to consider the intersecting effects of these discrimination types to better understand the complex mechanisms through which multiple discrimination affects labor allocation efficiency in the labor market.

Based on the argument above and the current situation of the labor market in China, we put forth the following proposition:

**Proposition:** No single best configuration of age, gender, hukou, educational background, and occupation that would lead to labor misallocation; rather, the cumulative interaction of multiple discrimination that would yield this outcome.

**Fig 1:**
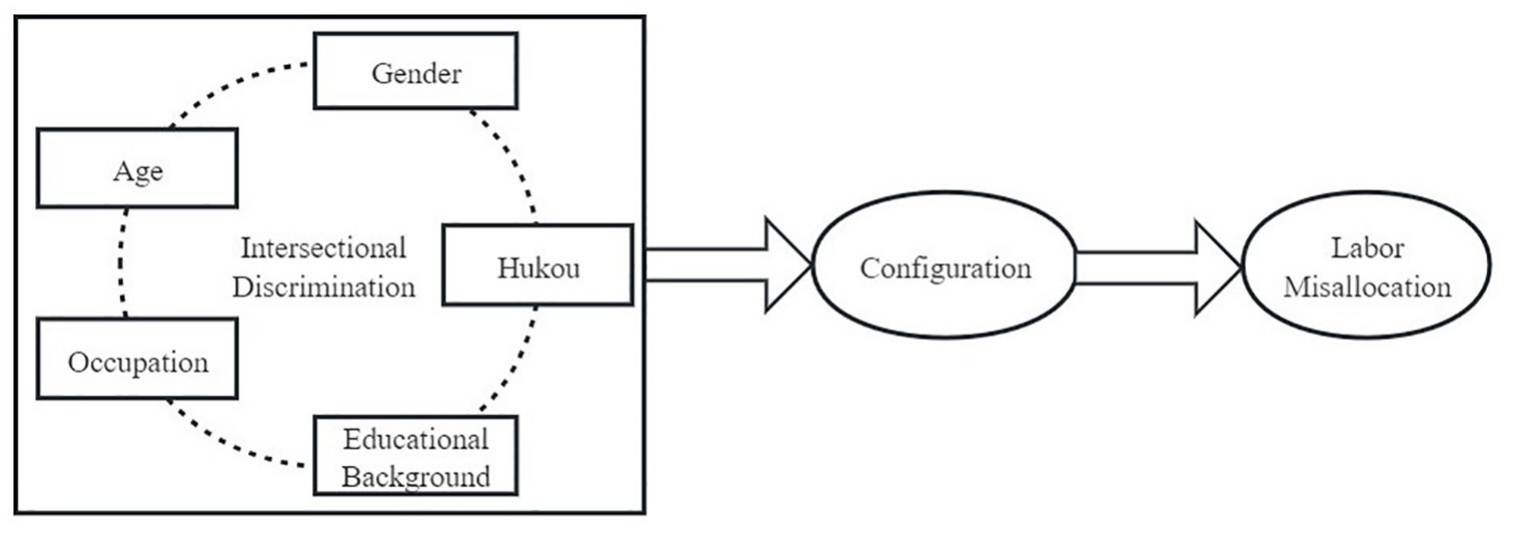
The framework of this research.

Taken together, these five forms of discrimination (age, gender, hukou, educational background, and occupation) may have complex synergistic effects in reducing the efficient allocation of labor factors. Based on this, we constructed a framework as shown in Fig 1.

## 3. Variables and methods

### 3.1. Data

The raw data in this paper come from the Chinese Social Survey (CSS) and the Statistical Yearbook of China. The CSS is a national survey launched in 2005 by the Institute of Sociology of the Chinese Academy of Social Sciences, including modules on employment, life and social attitudes. This article selects the latest data of CSS2021, which was themed “Social quality and modernization”. The raw data were processed as follows: (1) Samples with missing data of discrimination were removed. (2) Samples of age between ages 15 and 65 were retained. (3) CSS2021 were matched to regional data by province and samples with failed matches were deleted. Finally, we obtain cross-section data for a total of 8880 samples from 29 provinces in China for 2021. The advantage of using micro-survey data is that the volume of data not only meets the premise of a large sample and increases credibility, but also returns the question of the overall allocation of the labor resource to people-oriented.

### 3.2. Measures

#### 3.2.1. Labor misallocation (LMIS)

Referring to the research of Hsieh and Klenow (2009) [39], the article assumes that there is a distorted competitive market in the factor market, and defines the absolute distortion coefficient of labor factors as Formula (1):

$${LMIS}_{i}=\left(\frac{1}{\gamma_{i}}\right)-1$$ (1)

In the absence of resource misallocation, *LMIS*_*i*_ = 0. *LMIS*_*i*_≠0 means that the input price of labor factors is higher or lower than the average level, which will distort labor output. Under the premise of achieving the equilibrium of market competition, the relative distortion coefficient of labor is defined as *γ*_*i*_, as shown in Formula:

$$\gamma_{i}=\left(\frac{L_{i}}{L}\right)/\left(\frac{{S_{i}\beta }_{i}}{\sum_{i=1}^{N}{S_{i}\beta }_{i}}\right)$$ (2)

In practice, the absolute distortion coefficient of labor factors can be derived from the calculation of the relative distortion coefficient, which quantifies the degree of irrational labor distribution. Let *L*_*i*_ denote the labor input in region *i*, and *L* represent the national total labor input. The output value for region *i* is given by *S*_*i*_ *= Y*_*i*_ */Y*, where *Y*_*i*_ is the national output value. *Y*_*i*_ represents the national output value. *β*_*i*_ represents the weighted sum of regional output elasticity of labor. The elasticity of regional output with respect to labor, *β*_*i*_ represents the weighted sum of labor’s output elasticity in area *i*. The variable *γ*_*i*_ represents the theoretical labor ratio for region *i* under an optimal allocation scenario. If *γ*_*i*_ > 1, it indicates an over-allocation of labor resources in the region, as the actual labor input exceeds the theoretical value. Conversely, if *γ*_*i*_ < 1, it suggests an under-allocation of labor.

To estimate Eqs (1) and (2), the input-output elasticities for capital (*ɑ*_*i*_) and labor (*β*_*i*_) for each region must first be determined. We assume a Cobb-Douglas production function with constant returns to scale. And then using 2009 as the base period, the output variable *Y*_*i*_ is represented by the real GDP, labor input *L*_*i*_ by the number of employed individuals in each region, and capital input *K*_*i*_ by the fixed capital stock in each province. The depreciation rate is typically set at 9.6% in China. The labor misallocation index (*LMIS*_*i*_) is then calculated as the absolute value of the labor misallocation, with larger *LMIS*_*i*_ indicating a more severe misallocation of labor resources.

#### 3.2.2. Discrimination

Discrimination is measured by the level of perceived discrimination [40], which encompasses five specific dimensions: age, gender, hukou, educational background, and occupation discrimination. They were measured with five questions in the CSS2021 as follows: (1) Do you think the current situation of unfair treatment of age is serious? (2) Do you think the current situation of unfair treatment of gender is serious? (3) Do you think the current situation of unfair treatment of hukou is serious? (4) Do you think the current situation of unfair treatment of educational background is serious? (5) Do you think the current situation of unfair treatment of occupation is serious? There were five answer options for each question: ‘1-no problem’, ‘2-not too serious’, ‘3-relatively serious’, and ‘4-very serious’.

### 3.3. Method

#### 3.3.1. Fuzzy-set qualitative comparative analysis (fsQCA)

QCA is a method that examines the number and complexity of configural pathways between a set of predictor conditions and an outcome using set theory and Boolean logic [21,41]. This method has been used in fields including sociology, management, and political science. Compared with traditional regression methods that focus on "net effects", QCA is concerned with how antecedent conditions are combined or configured to produce specific outcomes [21,42]. The two principal aspects of Qualitative Comparative Analysis (QCA) are its ability to account for equifinality and to address causal asymmetry when examining the interplay of conditions. Equifinality denotes the scenario where several distinct combinations of conditions converge to produce an identical outcome. Meanwhile, causal asymmetry is the concept where the set of conditions that give rise to an outcome is separate from those that result in the absence of that outcome [41]. According to the data type, QCA can be divided into csQCA (crisp-sets QCA), mvQCA (multi value QCA), and fsQCA (fuzzy-sets QCA). FsQCA is suitable for dealing with three to eight continuous variables and considering the interdependence between conditions. fsQCA is preferred by researchers due to its higher precision in data calibration, which allows for any values between 0 and 1 instead of being limited to 0 or 1. It can more fully capture the subtle effects of variable changes in different degrees, better explain how multiple causes combine to produce equivalent results.

Because of these advantages and the complexity of multiple discrimination in the labor market, we selected this method to analyzes the configural pathways between causal conditions (here: multiple discrimination) and a result (here: labor misallocation) by calculating scores of necessity and sufficiency for each of the individual conditions and their combination effects [21].

#### 3.3.2. Calibration

The fsQCA commences with the calibration of scales, which requires a transformation of gathered data into fuzzy set membership, ranging from full non-membership (i.e., 0) to full membership (i.e., 1), with 0.5 demarcating the crossover point [21]. For discrimination calibration, we adopted the calibration approach for a 5-point Likert scale using 5, 3, and 1 as thresholds, as recommended by Pappas and Woodside (2021) [25]. To calibrate labor misallocation, we chose 0.95, 0.50, and 0.05 as the three thresholds, the data were converted into values between 0 and 1 [25].

#### 3.3.3. Model

This study intends to construct two models to analyze the data [43]. The outcome in the first model is the high-level labor misallocation. In the second model, the outcome is the non-high-level labor misallocation. It is important to consider both models because the asymmetric causality in fsQCA means that knowing the causes of a certain outcome does not imply that the causes of the opposite outcome are known. That is, a condition that leads to the outcome of interest does not mean that the opposite condition leads to the opposite outcome.

Model 1: LMIS = f (age; gender; hukou; educational background; occupation)
Model 2: ~LMIS = f (age; gender; hukou; educational background; occupation)

## 4. Results

### 4.1. Necessity analysis

Before the sufficiency analysis, it is necessary to examine whether a single condition can be a necessary condition for high and non-high-level labor misallocation. The results (Table 1) show that none of the conditions does not exceed the consistency threshold of 0.9 [21], indicating that each condition variable is not a necessary condition for regions to produce high-level labor misallocation.

**Table 1:** Analysis of necessary conditions.

Condition	Outcome: LMIS		Outcome: ~LMIS	
	Consistency	Coverage	Consistency	Coverage
Age	0.468	0.534	0.406	0.746
~Age	0.777	0.448	0.747	0.694
Gender	0.381	0.578	0.317	0.776
~Gender	0.852	0.436	0.828	0.683
Hukou	0.356	0.560	0.312	0.791
~Hukou	0.867	0.439	0.826	0.674
Educational background	0.487	0.501	0.452	0.749
~Educational background	0.756	0.461	0.699	0.687
Occupation	0.467	0.519	0.420	0.752
~Occupation	0.777	0.454	0.732	0.689

Note: ∼indicates the absence of or a low level.

### 4.2. Sufficiency analysis

We use fsQCA 3.0 to conduct sufficient conditional analysis. The case sample frequency threshold is set to 1, and the consistency threshold is set to 0.75 [21,25]. Considering recent methodological studies on QCA recommend reporting parsimonious solutions instead of intermediate solutions [44,45], Baumgartner and Thiem (2020) proved that only parsimonious solutions are guaranteed to be correct, this study reports parsimonious solutions to reduce the controversy of configuration results [44].

Table 2 shows the analysis result of sufficient conditions for Model 1 and Model 2. Sufficiency analysis identified five configurations for high-level labor misallocation (Model 2). In the configuration path, both the single path consistency and the overall solution consistency surpass the threshold of 0.75, and the overall solution coverage is 0.418, indicating that configuration can explain 41.8% of cases of high-level labor misallocation.

**Table 2:** Analysis of sufficient conditions for Models 1 and 2.

Type of discrimination	LMIS					~LMIS			
	1	2	3	4	5	1	2	3	4
Age	●	●	○		●				●
Gender			●	●	●	●			
Hukou	●	●	●				●		
Educational background		○		○	●			○	●
Occupation	○			●	○			●	
Raw coverage	0.248	0.243	0.235	0.249	0.244	0.317	0.312	0.260	0.305
Unique coverage	0.005	0.004	0.012	0.014	0.012	0.046	0.048	0.046	0.036
Consistency	0.792	0.802	0.805	0.804	0.816	0.776	0.791	0.832	0.788
Solution coverage	0.418					0.502			
Solution consistency	0.818					0.785			

Note: As per Fiss (2011) black circles “●” indicate the presence of antecedent conditions. White circles “○” indicate the absence or negation of antecedent conditions. Blank cells represent ambiguous conditions.

The first and second causal paths established by the fsQCA procedure indicate the joint power of age and hukou discrimination, suggesting that in the absence of occupational or educational background discrimination, the joint power of age and hukou discrimination can yield labor misallocation. The third configuration path reveals that, in the absence of age discrimination, gender and hukou discrimination can be sufficient for labor misallocation. The fourth causal path consists of the presence of gender and occupational discrimination with the absence of educational background discrimination. The fifth causal path identifies another distinct configurational setting for the presence of more kinds of discrimination. This path indicates that having serious discrimination of age, gender, and hukou, without occupational discrimination, can result in labor misallocation.

And there are four configuration paths for the absence of labor misallocation (Model 2): (1) serious gender discrimination; (2) serious hukou discrimination; (3) serious occupation discrimination without educational background discrimination; and (4) serious age and educational background discrimination. The coverage score for these pathways was 0.502, and the consistency score was 0.785.

### 4.3. Robustness test

This study adopted two methods to further verify the robustness of the results. One is to adjust the consistency threshold (from 0.75 to 0.78), the above configuration results did not change significantly. The other is to use the permutation test with the number of iterations of 10000 to test the possibility that QCA solutions may result from random chance [46]. Table 3 displays the permuted 95% confidence intervals for the consistency of the five pathways and the significance of the differences between the pathways. All 95% confidence intervals based on permutations included observed raw consistencies with all p-adj are 1. This suggests that the five configuration paths identified by QCA are significant differences and unlikely to result from random chance. Two pieces of evidence proved the robustness of the research results.

**Table 3:** Permutation tests for five configurations of LMIS.

Configuration paths	Consistency (observed)	95% CI of Consistency (permuted)	*p*-adj	se(*p*-adj)
1	0.732	[0.729, 0.756]	1	0
2	0.742	[0.738, 0.766]	1	0
3	0.755	[0.754, 0.778]	1	0
4	0.754	[0.746, 0.770]	1	0
5	0.756	[0.749, 0.773]	1	0

Note: The number of iterations is 10000. CI = confidence interval. The *p*-value adjustment method used was the Holm test.

## 5. Discussion

### 5.1. Configuration paths analysis

Under the framework of intersectionality, this study is the first to examine the differentiated configuration paths of various types of employment discrimination that contribute to labor misallocation through the fsQCA method. The results show that: a single condition of discrimination is difficult to form high labor misallocation. Among the different configurations of six types of discrimination, there are five pathways to form high labor misallocation, which can be classified into four interaction modes: age-hukou, gender-hukou, gender-occupation, and age-gender-educational background. The results reflect the central principles of intersectionality theory, which posits that the overlapping and interaction of various forms of discrimination can lead to the emergence of distinct and intricate social dynamics [13–16]. Our proposition was generally supported.

Furthermore, compared with the four paths for non-high labor misallocation, the configuration paths for high labor misallocation are characterized by the interaction of different discrimination forms. Specifically, the interaction of at least two kinds of discrimination could result in high-level labor misallocation, which could not occur when only one kind of discrimination is present. This finding partially reflects the insufficiency of the logic behind previous studies that used traditional linear analysis to investigate a single form of discrimination and underscores the necessity for a complex perspective to analyze multiple discrimination [24]. This paper, building on intersectionality theory, substantiates the perspective that the interplay of multiple forms of discrimination impacts the overall performance of the labor market (labor misallocation) and uncovers potential configurational pathways.

Besides, in the five configuration paths of high labor misallocation, each of the discrimination forms of age, gender, and hukou plays an important role in three of these paths, but the discrimination of educational background and occupation only affects one of them. This indicates that the discrimination of age, gender, and hukou is more serious compared to discrimination of educational background and occupation. As indicated by the Global Sustainable Development Report 2023 and the Global Report on Ageism, gender and age discrimination remain significant issues faced by countries worldwide, and such issues have been confirmed within China’s labor market. Because of China’s hukou system, migrant workers have limited access to various public services in urban areas. Despite certain advancements in mitigating hukou discrimination as a result of continuous reforms to the hukou system in recent years [47], it continues to be a major form of discrimination impeding the efficient distribution of labor resources in China [48]. Consequently, accelerating the reform of the hukou system is imperative, as it is vital for eradicating the institutional discrimination experienced by migrant workers and for diminishing the adverse cumulative impacts that may stem from such discrimination. Relative to other types, discrimination of educational background and professional discrimination is less significant and requires interaction with gender discrimination to exert affects. In line with this, women were particularly discriminated against in connection with jobs that involved mixed occupations, and in jobs requiring both high and low education levels [49]. This collectively illustrates that within the labor market, some forms of discrimination are likely more deeply ingrained and widespread than others, shaped by the long-standing influences of the social fabric and cultural backdrop of the region.

### 5.2. Contributions

This study has made several contributions to research on discrimination and labor misallocation. First, this study expands the research literature on discrimination and labor allocation in the labor market. Unlike many previous studies that focused on exploring the net effects of various potential factors on labor misallocation, this study goes beyond the net effect approach [25,41]. It adopts a complexity perspective to explore the causal mechanisms underlying the insufficient efficiency of labor allocation in the context of multiple discrimination in the labor market. It reveals the configuration pathways involved, providing a deeper understanding of the interaction effects [25]. Second, the research extends the application of intersectionality theory by combining it with the fuzzy-set Qualitative Comparative Analysis (fsQCA) method, which is also based on the complex relationships between variables. The fsQCA method allows for the examination of the impact process of multiple, interrelated factors on labor allocation efficiency, thereby providing an empirical test for the intersectionality framework [13,16]. This integrated approach provides a novel perspective on the impact of multiple discrimination on labor allocation efficiency and offers new insights for related studies.

## 6. Conclusions and implications

This study constructs a theoretical framework of the impact of multiple discriminations on labor allocation efficiency based on intersectionality theory, and uses the fsQCA method to identify the potential configuration paths of six forms of discrimination on labor allocation efficiency. Using the data from the Chinese Social Survey 2021 and the Statistical Yearbook of China, the main research conclusions are as follows: from a single form discrimination standpoint, none of the five forms of discrimination can be deemed a necessary condition for achieving high-level labor misallocation. Among the different configurations of six types of discrimination, there are five pathways to form high-level labor misallocation, which can be classified into four interaction modes: age-hukou, gender-hukou, gender-occupation, and age-gender-educational background. And there are four configuration paths for the absence of labor misallocation. In comparation, the discrimination of age, gender, and hukou are more severe issues than educational background and occupational discrimination.

This study has some limitations. First, only five types of discrimination were selected to analysis as the condition variables, this could mean other types of discrimination were missed (e.g., beauty or weight). Future research can collect more date of discrimination to analysis the interaction effects on labor misallocation. Second, the dataset used in this research are cross-sectional data from a single time, which lacks cross-year case data. An important next step may also be to collect multi-year data and combine other methods to judge the long-term impact of multiple discrimination on labor misallocation. In addition, the research samples selected in this study are concentrated in China, where regional differences in discrimination types and labor misallocation levels are present, subsequent studies might assess the generalizability of our findings to other regions.

Moreover, this study provides practical implications for Chinese government officials and policymakers. Firstly, the government should recognize the intersectionality of discrimination and prioritize addressing age, gender, and hukou discrimination. Therefore, policymakers should launch anti-discrimination policies based on intersectionality to provide more realistic legal protection for workers. And the government should improve the recruitment mechanism of enterprises and industries, protect the legitimate rights and interests of workers. Concurrently, the government should proactively implement labor training programs, aiming to enhance the knowledge and skill levels of the workforce, thereby reducing the level of discrimination faced by the workers and improving labor productivity efficiency. Secondly, employers should actively create a fair employment environment with equal job opportunities and deserved wages for workers, and increase personal output by stimulating the enthusiasm of workers, so as to achieve a better overall efficiency of labor allocation. Besides, employers could collaborate with social organizations to provide psychological counseling, skills training, and employment assistance services for workers to minimize the cumulative effect of the interaction of discrimination.
